# Acute coronary occlusion among cancelled STEMI alerts: a prospective study in a telemedicine-guided network

**DOI:** 10.1093/ehjdh/ztag012

**Published:** 2026-02-05

**Authors:** Blanca Herrera, David García, Anna Cufí, Aida Feu, Simon Tapia, Carmen Martín, Victor Agudelo, Pablo Loma-Osorio, Rafel Ramos, Ramon Brugada, Jaime Aboal

**Affiliations:** Department of Cardiology, Hospital Universitari Dr Josep Trueta, Avinguda de França s/nd, Girona 17007, Spain; Department of Cardiology, Hospital Universitari Dr Josep Trueta, Avinguda de França s/nd, Girona 17007, Spain; Department of Cardiology, Hospital Universitari Dr Josep Trueta, Avinguda de França s/nd, Girona 17007, Spain; Department of Cardiology, Hospital Universitari Dr Josep Trueta, Avinguda de França s/nd, Girona 17007, Spain; Department of Cardiology, Hospital Universitari Dr Josep Trueta, Avinguda de França s/nd, Girona 17007, Spain; Department of Cardiology, Hospital Universitari Dr Josep Trueta, Avinguda de França s/nd, Girona 17007, Spain; Department of Cardiology, Hospital Universitari Dr Josep Trueta, Avinguda de França s/nd, Girona 17007, Spain; Department of Cardiology, Hospital Universitari Dr Josep Trueta, Avinguda de França s/nd, Girona 17007, Spain; ISV Research Group, Primary Care Services, Institut Universitari d’Investigació en Atenció Primaria Jordi Gol (IDIAP J Gol), Catalonia 08007, Spain; Cardiovascular Genetics Centre, Biomedical Research Institute, Girona 17190, Salt, Spain; Medical Science Department, School of Medicine, University of Girona, Girona 17071, Spain; Department of Cardiology, Hospital Universitari Dr Josep Trueta, Avinguda de França s/nd, Girona 17007, Spain; Cardiovascular Genetics Centre, Biomedical Research Institute, Girona 17190, Salt, Spain; Medical Science Department, School of Medicine, University of Girona, Girona 17071, Spain; Centre for Biomedical Research in Cardiovascular Diseases Network, Madrid 28029, Spain; Department of Cardiology, Hospital Universitari Dr Josep Trueta, Avinguda de França s/nd, Girona 17007, Spain; Cardiovascular Genetics Centre, Biomedical Research Institute, Girona 17190, Salt, Spain

**Keywords:** STEMI networks, Acute coronary occlusion, Telematic evaluation, Cancelled STEMI alerts, ECG interpretation, Digital health platform

## Abstract

**Aims:**

ST-segment elevation myocardial infarction (STEMI) networks often face false-positive activations, leading to unnecessary catheterization laboratory use. To optimize resource allocation, STEMI alerts are sometimes cancelled after telematics evaluation; however, this strategy may result in missed cases of acute coronary occlusion (ACO) requiring urgent revascularization.

**Methods and results:**

This prospective, single-centre study included patients with initially activated but subsequently cancelled STEMI alerts via the ODISEA digital platform between January 2022 and December 2024. Based on coronary angiography, patients were classified as having ACO (TIMI 0–1 flow with thrombotic appearance) or no occlusion. Baseline characteristics, electrocardiogram (ECG) findings, angiographic data, and in-hospital mortality were compared. Of 2259 STEMI activations, 665 alerts (29.4%) were cancelled following a telematic assessment. Among these, 28 patients (4.2%) had ACO. Compared to the remaining cohort, they had higher rates of hypertension (78.6% vs. 60.3%; *P* = 0.03), diabetes (46.4% vs. 28.4%; *P* = 0.03), and prior coronary artery bypass grafting (10.7% vs. 2.5%; *P* = 0.01). Predominant ECG findings included <1 mm ST-segment elevation (67.8%) and ST-segment depression (25%). The left anterior descending artery was most frequently involved. In-hospital mortality was 10.7% in the ACO group and 7.7% in the non-ACO group (*P* = 0.50).

**Conclusion:**

Among cancelled STEMI alerts, missed ACO cases were infrequent, often presenting with subtle or non-classical ECG findings. These patients showed a higher burden of cardiovascular risk and increased in-hospital mortality.

## Graphical Abstract

**Graphical Abstract:**
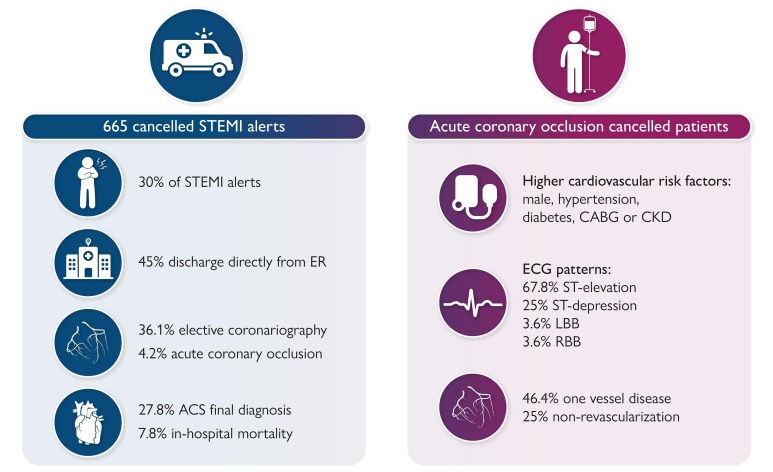

## Introduction

A major challenge in ST-segment elevation (STE) myocardial infarction (STEMI) networks is the high rate of false-positive activations, in which patients initially diagnosed with STEMI are later found not to have the condition. These inappropriate activations may interfere with the elective workflow of the catheterization laboratory, reducing efficiency and necessitating unplanned reorganization of scheduled procedures, and may potentially affect the timely management of patients who truly require urgent reperfusion. Previous studies estimate that between 14% and 36% of STEMI activations result in non-acute coronary syndrome (ACS) diagnoses, contributing to resource strain and possible delays in reperfusion therapy.^1–3^

Digital health platforms have emerged as valuable tools to support real-time data sharing and improve decision-making. By enabling rapid access to clinical data and remote interpretation of electrocardiograms (ECGs), these systems can reduce unnecessary transfers for primary percutaneous coronary intervention (PCI) and enhance the efficiency of STEMI networks. Studies have demonstrated that such platforms can decrease the proportion of patients transferred with non-ACS diagnoses and improve time-to-reperfusion metrics.^4–6^

Despite these advancements, patients whose STEMI alerts are cancelled prior to hospital transfer remain an understudied population. These patients often present with multiple comorbidities and atypical or non-diagnostic ECG patterns and may experience higher in-hospital mortality compared to those who proceed directly to PCI.^7,8^ Recent evidence suggests that up to 30% of STEMI alerts are cancelled following remote multidisciplinary assessment, with many of these patients ultimately diagnosed with alternative life-threatening conditions such as sepsis, aortic dissection, or pulmonary embolism.^9^

The objective of this study was to determine the proportion of patients with an acute coronary occlusion (ACO) among those whose STEMI alerts were cancelled following telematic evaluation. Additionally, we aimed to compare the clinical, electrocardiographic, angiographic, and in-hospital mortality profiles of these patients with those of the remaining cancelled population.

## Methods

### Study design

This is a prospective, observational, single-centre study conducted between 1 January 2022 and 31 December 2024. The study has been approved by the institutional ethics committee and complies with the principles of the Declaration of Helsinki.

### Study population

The study included patients for whom a STEMI alert was initially activated but later cancelled following a multidisciplinary decision involving the first medical contact (FMC) and the on-call cardiologist, using the ODISEA digital health platform. Inclusion criteria were cancelled STEMI alerts after real-time telematic assessment and availability of clinical and ECG data at the time of activation. Exclusion criteria included direct transfer to a catheterization laboratory without cancellation and cases of clearly inappropriate alert activation, identified during cardiology review of ECGs. These comprised patterns unrelated to STEMI suspicion, such as conduction abnormalities without STE or supraventricular arrhythmias without ischaemic changes.

ODISEA platform and decision-making process: ODISEA is a digital platform designed to improve STEMI triage by enabling real-time sharing of clinical data, ECG images, and decision-making among healthcare professionals. Its main functionalities have been previously described.^4^ Prior to deployment, all professionals participating in the STEMI network received specific training to activate alerts for both classic STE and ECG patterns suspicious for acute coronary occlusion without ST elevation, such as posterior ischaemia, de Winter pattern, hyperacute T waves, or new conduction abnormalities (LBBB, RBBB, or paced rhythms).

Upon alert activation, the FMC uploads patient demographics, clinical information, ECG photographs, and prehospital treatment data. The on-call cardiologist and the interventional team via a secure communication system instantly review these. The decision to proceed with urgent transfer for primary angioplasty was based on consensus among the FMC, the on-call cardiologist, and the interventional cardiologist. In the absence of consensus, the interventional cardiologist had the final authority. If transfer was deemed unnecessary, the alert was cancelled, and the patient was redirected to the nearest emergency department for further evaluation. In the emergency department, after a complete clinical, electrocardiographic, and laboratory assessment (complemented by imaging studies when indicated) patients followed one of the three management pathways: (1) discharge directly from the emergency department after the safe exclusion of relevant disease, including ACS; (2) hospital admission to the cardiology department when a cardiac diagnosis was suspected; or (3) admission to other specialties based on the suspected diagnosis. No patient was discharged directly from the emergency department after undergoing coronary angiography. Importantly, decision to perform coronary angiography after cancellation was made at the discretion of the attending cardiologist based on clinical judgment.

### Patient classification

Patients who underwent coronary angiography after alert cancellation were classified into two groups according to their angiographic findings: The ACO group, defined by the presence of TIMI 0–1 flow and a thrombotic appearance in a culprit artery, and the non-ACO group, comprising cancelled patients who did not meet these criteria.

### Study endpoints

The primary objective of the study was to determine the proportion of ACO among patients with cancelled STEMI alerts and to compare their clinical characteristics, ECG findings, angiographic features, and in-hospital mortality with those of the remaining cancelled population. Secondary objectives included the following: a detailed description of the ECG patterns and the relationship between ECG findings and angiographic results in ACO patients.

### Data collection

Demographic data, medical history, and cardiovascular risk factors were collected. The type of FMC was documented, including emergency medical services (EMS), emergency departments at non-PCI-capable hospitals, and primary care physicians. ECGs were retrospectively reviewed by five experienced cardiologists. Classification was based on the initial ECG transmitted at the time of alert activation and the discussion between FMC and the on-call cardiologist. ECG patterns were categorized as STE, ST-segment depression, T-wave abnormalities (inverted or hyperacute), left bundle branch block (LBBB), right bundle branch block (RBBB), pacemaker rhythm, and Q waves. Angiographic variables included the presence and location of coronary lesions, initial TIMI flow, and need for revascularization via PCI or coronary artery bypass grafting (CABG). Final diagnoses were obtained from official hospital discharge summaries.

#### Statistical analysis

Continuous variables were assessed for normality using the Kolmogorov–Smirnov test. Normally distributed variables are reported as mean ± standard deviation (SD), while non-normally distributed variables are presented as median and interquartile range (IQR). Categorical variables are expressed as absolute numbers and percentages.

Comparisons between groups were performed using the independent samples *t*-test for normally distributed continuous variables and the Mann–Whitney U test for non-normally distributed variables. The chi-square test was used for categorical data when the expected cell counts were adequate; otherwise, Fisher’s exact test was applied.

*P* values are reported with two decimal places, except those <0.001 that are reported as ‘<0.001’. A *P* value of <0.05 was considered statistically significant.

No adjustment for confounding was performed due to the limited sample size of the acute coronary occlusion group. Analyses were conducted using SPSS version 24.0 (IBM, Chicago, IL, USA).

## Results

**Study population and baseline characteristics:**

**Figure 1:**
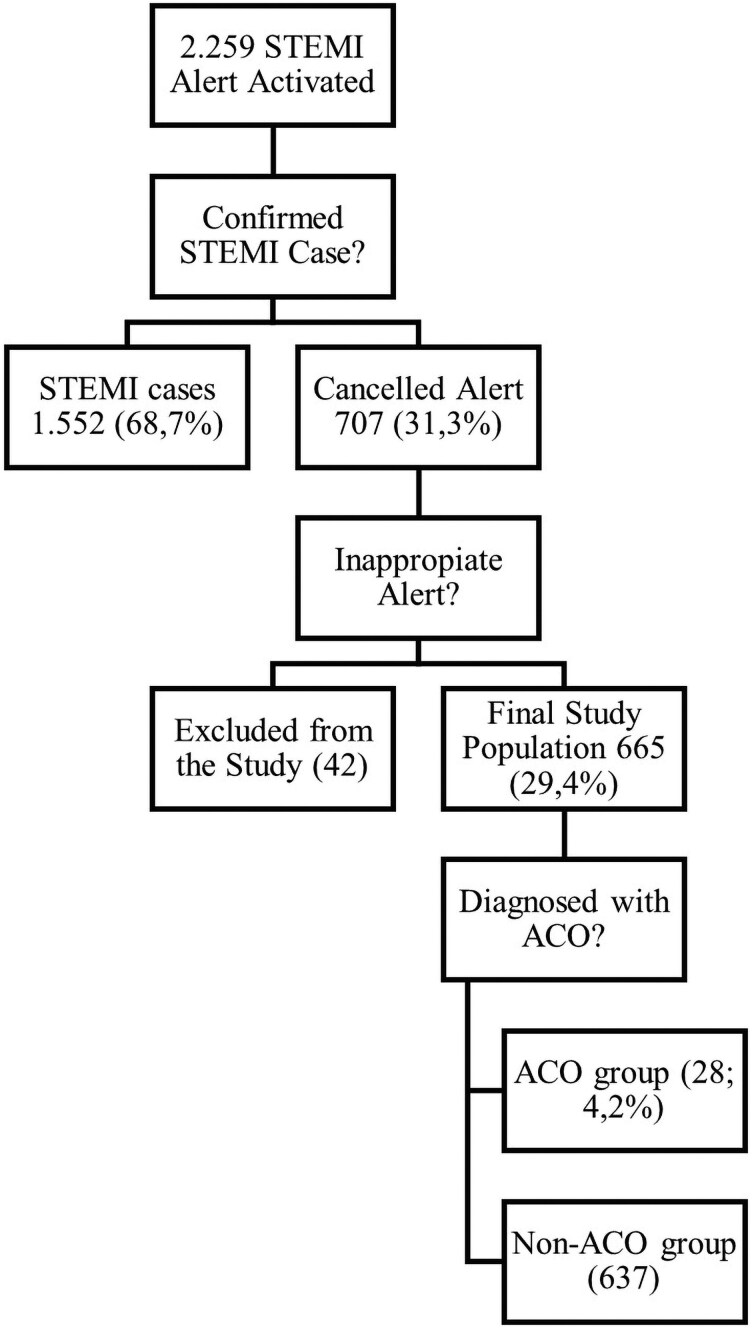
STEMI alert process flowchart. STEMI, ST-segment elevation myocardial infarction; ACO, acute coronary occlusion.

During the study period, a total of 2259 patients had a STEMI alert activated. Of these, 1552 (68.7%) were confirmed STEMI cases and treated accordingly. The remaining 707 alerts (31.3%) were cancelled following a telematic assessment. After excluding 42 patients due to inappropriate alert activation (ECG patterns clearly inconsistent with STEMI alerts), the final study population comprised 665 cancelled patients (29.4% of all STEMI alerts). Among them, 28 patients (4.2%) were diagnosed with an ACO based on coronary angiographic findings, while 637 had no evidence of occlusion and were included in the non-ACO group. The study flow chart is shown in *Figure 1*.

**Figure 2:**
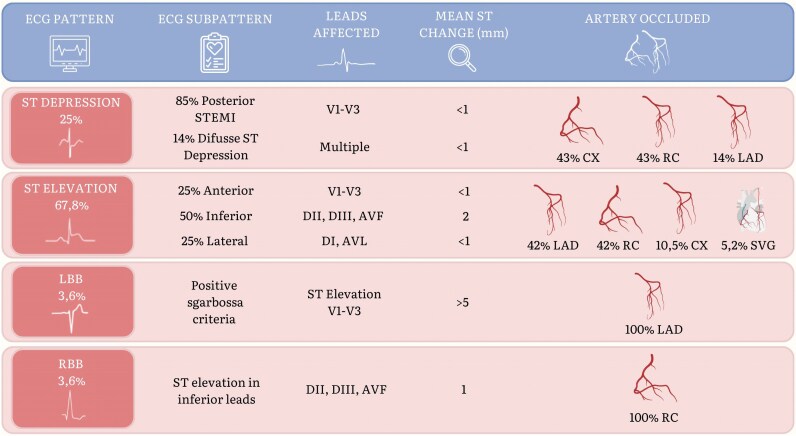
Electrocardiographic patterns and angiographic correlations in patients with acute coronary occlusion. Summary of ECG patterns, subpatterns, key findings, and corresponding culprit arteries in patients with acute coronary occlusion. ST depression was mainly associated with posterior infarction and circumflex or posterolateral artery occlusion. ST elevation patterns included anterior (LAD), inferior (RC, CX, SVG), and lateral (DG, CX) territories. LBBB fulfilled Sgarbossa criteria, and RBBB was linked to inferior STEMI. Submillimetric ST elevation in inferior leads was present in 67.8% of cases.

Baseline characteristics for the overall cohort and both subgroups are summarized in *Table 1*. Compared with the non-ACO group, ACO patients had a significantly higher prevalence of hypertension (78.6% vs. 60.3%; *P* = 0.03), diabetes mellitus (46.4% vs. 28.4%; *P* = 0.03), prior PCI (25.0% vs. 12.6%; *P* = 0.05), chronic kidney disease (28.6% vs. 14.0%; *P* = 0.04), and previous CABG (10.7% vs. 2.5%; *P* = 0.01). They were also more frequently male (85.7% vs. 68.8%; *P* = 0.04).

**Table 1:** Baseline characteristics, cardiovascular risk factors, electrocardiographic features, discharge diagnosis, and in-hospital mortality among the overall cohort, patients with acute coronary occlusion (ACO group), and cancelled patients without ACO (non-ACO group)

Variables	Overall (665)	ACO group (28)	Non-ACO group (637)	*P*
Age. median/SD	67 (16)	68 (11)	67 (16)	0.6
Women *n*%	203 (30.5%)	4 (14.3%)	199 (31.2%)	**0.04**
Smoker	172 (25.9%)	6 (21.4%)	166 (26.1%)	0.5
Hypertension	406 (61.1%)	22 (78.6%)	384 (60.3%)	**0**.**03**
Diabetes	194 (29.2%)	13 (46.4%)	181 (28.4%)	**0**.**03**
Dyslipidaemia	321 (48.3%)	16 (57.1%)	305 (47.9%)	0.3
Previous stroke	58 (8.7%)	0	58 (9.1%)	0.09
Previous AMI	126 (18.9%)	9 (32.1%)	117 (18.4%)	0.06
Previous PCI	87 (13.1%)	7 (25%)	80 (12.6%)	0.05
Previous CABG	19 (2.9%)	3 (10.7%)	16 (2.5%)	**0**.**01**
COPD	66 (9.9%)	2 (7.1%)	64 (10%)	0.6
Chronic kidney disease	101 (15.2%)	8 (28.6%)	93 (14%)	**0**.**04**
OHCA	17 (2.6%)	1 (3.6%)	16 (2.5%)	0.7
First medical contact:				0.5
Home/public place	174 (26.2%)	5 (17.9%)	169 (26.5%)	
Hospital without cathlab	340 (51.1%)	16 (57.1%)	324 (50.9%)	
Primary care centre	151 (22.7%)	7 (25%)	144 (22.6%)	
Discharge from emergencies room	299 (45%)	0	299 (46.9%)	**<0**.**001**
Pattern ECG STEMI alert:				0.1
ST elevation	293 (44.1%)	19 (67.8%)	277 (43.5%)	
ST depression	142 (21.4%)	7 (25%)	135 (21.2%)	
LBBB	83 (13%)	1 (3.6%)	84 (12.6%)	
RBBB	48 (7.5%)	1 (3.6%)	49 (7.4%)	
Pacemaker	25 (3.8%)	0	25 (3.9%)	
T waves changes	56 (8.4%)	0	53 (8.3%)	
Q waves	16 (2.4%)	0	16 (2.5%)	
Coronary angiography	240 (36.1%)	28 (100%)	212 (33.3%)	**<0**.**001**
Acute coronary syndrome	185 (27.8%)	28 (100%)	157 (24.6%)	**<0**.**001**
In-hospital mortality	52 (7.8%)	3 (10.7%)	49 (7.7%)	0.5

ACO, acute coronary occlusion; AMI, acute myocardial infarction; CABG, coronary artery bypass grafting; COPD, chronic obstructive pulmonary disease; ECG, electrocardiogram; ED, emergency department; FMC, first medical contact; LBBB, left bundle branch block; NSTEMI, non-ST-elevation myocardial infarction; OHCA, out-of-hospital cardiac arrest; PCI, percutaneous coronary intervention; RBBB, right bundle branch block; SD, standard deviation; STEMI, ST-elevation myocardial infarction. Bold values indicate statistical significance.

There were no statistically significant differences in ECG patterns prompting STEMI alert activation although STE was numerically more frequent in the ACO group (57.1% vs. 43.5%; *P* = 0.10).

In-hospital mortality for the entire study population was 7.8% (52 patients), with no statistically significant difference between the ACO and non-ACO groups (10.7% vs. 7.7%; *P* = 0.50). Causes of death included cardiogenic shock (*n* = 23), neurological events (*n* = 9), malignancy (*n* = 4), sepsis (*n* = 3), respiratory failure (*n* = 2), aortic dissection (*n* = 2), pulmonary embolism (*n* = 1), and unknown causes (*n* = 7).

A large proportion of patients were discharged directly from the emergency department, accounting for 45.0% of the entire cohort. This was observed exclusively in the non-ACO group (46.9% vs. 0%; *P* < 0.001).

Final diagnoses among the full cohort included NSTEMI (26.8%) and non-cardiac chest pain (23.5%) as the most frequent. Among ACO patients, only 25.0% were discharged with an STEMI diagnosis, while 75.0% were classified as NSTEMI despite confirmed occlusion. In contrast, non-ACO patients were most diagnosed with non-cardiac chest pain (24.5%), NSTEMI (24.6%), or other non-ischaemic conditions such as arrhythmias (10.5%), acute heart failure (7.8%), and pericarditis (6.9%). None in the non-ACO group received a final STEMI diagnosis (*P* < 0.001).

**Angiographic findings:**

Coronary angiography was performed in 240 patients (36.1% of the overall study population), including all cases in the ACO group (*n* = 28) and 212 patients (33%) from the non-ACO group. The remaining 425 patients (63.9%), all belonging to the non-ACO group, were managed without coronary angiography. In 73.3% of cases, the procedure was conducted within the first 24 h following the initial STEMI alert. The remaining procedures were performed between days 2 and 4, based on clinical re-evaluation by the attending cardiologist. Angiographic findings are summarized in *Table 2*.

**Table 2:** Coronary angiographic findings and revascularization strategies among patients undergoing catheterization, comparing those with acute coronary occlusion (ACO) and cancelled patients without occlusion

Variable	Overall (240)	ACO group (*n* = 28)	Non-ACO group (212)	*P*
No lesions	98 (40.8%)	0	98 (46.2%)	**<0.001**
One vessel disease	71 (29.6%)	13 (46.4%)	58 (27.4%)	
Two-vessel disease	26 (10.8%)	10 (35.7%)	16 (7.5%)	
Three-vessel disease	36 (15%)	5 (17.9%)	31 (14.6%)	
Left main	9 (3.8%)	0	9 (4.2%)	
TIMI flow:				**<0**.**001**
TIMI 0	34 (14.2%)	23 (82.1%)	11 (5.2%)	
TIMI 1	5 (2.1%)	5 (17.9%)	0	
TIMI 2	15 (6.3%)	0	15 (7.1%)	
TIMI 3	186 (77.5%)	0	186 (87.7%)	
TIMI flow 0–1:				**<0**.**001**
Chronic	11 (4.5%)	0	11 (5.1%)	
Acute	28 (11.6%)	28 (100%)	0	
Revascularization treatment:				**<0**.**001**
PCI	89 (37.1%)	20 (71.4%)	69 (32.5%)	
CABG	17 (7.1%)	1 (3.6%)	16 (7.5%)	
Fail PCI	5 (2.1%)	4 (14.3%)	1 (0.5%)	
No revascularization	129 (53.8%)	3 (10.7%)	126 (59.4%)	

ACO, acute coronary occlusion; CABG, coronary artery bypass grafting; PCI, percutaneous coronary intervention; TIMI, thrombolysis in myocardial Infarction. Bold values indicate statistical significance.

Among patients undergoing catheterization, 40.8% presented with no significant coronary lesions, while 29.6% had single-vessel disease, 10.8% had two-vessel disease, 15.0% had three-vessel disease, and 3.8% had left main coronary artery disease. In the ACO group, single-vessel disease predominated (46.4%), followed by two-vessel (35.7%) and three-vessel disease (17.9%). In contrast, among the 212 non-ACO patients who underwent angiography, 46.2% showed no significant coronary stenosis.

In the non-ACO group, initial TIMI 0 flow was observed in only 5.2% of cases, all of which were classified as chronic total occlusions without angiographic features of acute thrombosis. Conversely, TIMI 3 flow was the predominant finding, present in 87.7% of patients.

Regarding revascularization, 71.4% of patients in the ACO group underwent PCI, compared to 32.5% of non-ACO patients (*P* < 0.001). The need for CABG was low and similar between groups (3.6% in ACO vs. 7.5% in non-ACO; *P* = NS). PCI failure was significantly more frequent in the ACO group (14.3% vs. 0.5%; *P* < 0.001) due to the thrombotic burden of acute occlusion lesions.

Among the 425 patients who did not undergo coronary angiography, the final discharge diagnoses were as follows: non-specific chest pain in 206 patients (48.5%), acute heart failure in 35 (8.2%), pericarditis in 35 (8.2%), aortic dissection in 4 (0.9%), pulmonary embolism in 10 (2.4%), sepsis in 28 (6.6%), non-ST-elevation ACS in 9 (2.1%), and other causes in 98 (23.1%).

**Electrocardiographic patterns and angiographic correlations in patients with acute coronary occlusion:**

Among the 28 patients diagnosed with acute coronary occlusion, electrocardiographic presentations were highly variable and frequently deviated from classical STEMI patterns. A detailed summary of ECG subtypes, associated coronary lesions, and angiographic correlations is provided in *Figure 2*.

The predominant ECG finding was STE, present in 67.8% of cases. Among these, the distribution was anterior (25%), inferior (50%), and lateral (25%). All anterior STEMI patterns were associated with occlusion of the left anterior descending (LAD) artery. Inferior STEMI was most related to right coronary artery (RCA) occlusion (80%), followed by the circumflex artery (10%) and saphenous vein grafts (10%). Lateral STEMI patterns were associated with occlusions in diagonal branches (60%) and the circumflex artery (40%).

ST-segment depression was identified in 25% of ACO cases. Among these, 85% corresponded to isolated posterior STEMI, while the remaining 15% showed diffuse ST depression patterns. In patients with posterior STEMI, the culprit vessel was the circumflex artery in 50% of cases, followed by posterolateral branches (33%) and the RCA (17%). Diffuse ST depression was exclusively observed in patients with LAD occlusion (100%).

Left bundle branch block was identified in one patient and was accompanied by positive Sgarbossa criteria. RBBB was also observed in one of the patients and showed an inferior STE, corresponding to RCA occlusion.

Finally, submillimetric (<1 mm) ST elevation was identified in 67.8% of patients, particularly in inferior leads (DII, DIII, and aVF).

**ODISEA telemedicine platform performance metrics:**

The median time between diagnostic ECG acquisition and initiation of remote cardiology evaluation was 4.98 (IQR 0.98–13.56) min. The platform was used in 92% of all STEMI activations, indicating high adherence to the telemedicine workflow. In all cancelled alerts, a structured final report was automatically generated, detailing the reason for cancellation and the presumptive diagnosis established in the emergency department, and transmitted to the FMC.

## Discussion

The present study demonstrates the effective and safe use of telemedicine to support clinical decision-making within a regional STEMI network. The implementation of the ODISEA platform enabled remote and rapid evaluation by cardiologists, improving the interpretation of complex or uncertain ECG patterns and thereby reducing unnecessary patient transfers (29.4% of STEMI alerts were cancelled after remote evaluation). A small proportion of cancelled patients (4.2%) were later found to have an ACO during non-urgent angiography. These patients shared a common clinical profile characterized by a higher burden of cardiovascular risk factors or previous cardiac disease, and by subtle ECG abnormalities such as submillimetric STE or ST-segment depression.

STEMI alert cancellations are a frequent and necessary component of modern STEMI networks, aiming to prevent unnecessary catheterization laboratory activations and optimize resource allocation. In our cohort, 29.4% of STEMI alerts were cancelled following real-time multidisciplinary evaluation via a telemedicine platform. This cancellation rate falls within the wide range reported in the literature, spanning from 7.6% to over 50%, depending on the study population and inclusion criteria.^1–3,10,11^ Variability may stem from differences in platform availability, triage protocols, degree of consensus decision-making, and whether only patients arriving at PCI-capable centres are included. Standardization of alert activation and cancellation protocols would facilitate more accurate inter-study comparisons and benchmarking of STEMI network performance.

Telemedicine platforms offer valuable support for improving triage accuracy in prehospital STEMI care. In our region, the use of ODISEA led to a reduction in unnecessary transfers and a 7.6% decrease in non-ACS patients undergoing catheterization.^4^ These findings are in line with data from other multidisciplinary platforms, which have shown benefits in minimizing reperfusion delays and improving case selection.^5,12^ Moreover, as demonstrated in our study, telemedicine allows the safe and efficient cancellation of urgent activations to the catheterization laboratory after real-time expert evaluation. This effectiveness is reflected in the short median response time from diagnostic ECG acquisition to remote evaluation (4.98 min), the high rate of platform use driven by the need to support FMCs in interpreting complex ECGs, and the automatic feedback reports generated for each case, providing continuous communication and learning. The recent consensus document by the Society for Cardiovascular Angiography and Interventions endorses the integration of real-time telemedicine tools into STEMI workflows.^13^

The decision to cancel an alert carries inherent clinical risk, particularly in overlooking patients with an ACO requiring urgent revascularization. In our study, 4.2% of the cancelled patients were later diagnosed with ACO. This rate is lower than those reported by Faour *et al*. (13%) and Lange *et al*. (10%),^7,8^ potentially reflecting differences in network configuration, use of structured telematic tools, and access to expert ECG interpretation. Importantly, the ACO subgroup had a higher prevalence of cardiovascular risk factors, including hypertension, diabetes, and prior coronary revascularization findings consistent with previous reports.^8,14^ Taken together, these results indicate that while ECG patterns alone did not distinguish ACO from non-ACO patients, the burden of cardiovascular risk provided an additional discriminative value, suggesting that integrating clinical profiles into cancellation decisions may improve safety and reduce missed occlusions.

The traditional reliance on STE as the primary criterion for STEMI activation is being increasingly challenged. In our study, 25% of ACO patients presented with ST-segment depression rather than elevation and 67.8% had ST elevation (<1 mm), underscoring the limitations of conventional thresholds. The occlusion myocardial infarction paradigm proposes a shift from STE-based classifications to a pathophysiologically driven model centred on acute vessel occlusion, regardless of ECG morphology. This approach is supported by recent findings indicating that up to 30% of NSTEMI presentations may harbor undetected ACOs, with worse outcomes due to delayed intervention.^15^ These complex presentations highlight the potential value of telemedicine systems that integrate previous ECGs and structured comorbidity data to facilitate expert interpretation and reduce diagnostic misclassification.^16^

Finally, emerging innovations such as artificial intelligence (AI) offer promising opportunities to support ECG interpretation. AI-enhanced models have demonstrated superior diagnostic performance compared with conventional criteria in detecting ACO ECG patterns.^6,17–19^ These technologies promise faster and more accurate identification of ACO, particularly in cases with atypical or subtle ECG changes.

This study has several limitations. First, it was a single-centre observational analysis conducted within a specific regional STEMI network, which may limit the generalizability of the findings to other healthcare systems with different telemedicine infrastructures, alert activation protocols, or resource availability. In addition, the relatively small number of patients in the ACO group may limit the statistical power of some analyses and the strength of subgroup comparisons. Second, although ECGs were reviewed by experienced cardiologists, the interpretation of subtle or non-classical patterns is inherently subjective and may have introduced classification bias. Third, coronary angiography was not systematically performed in all patients but only when considered clinically necessary by the attending physicians, and both admission and invasive testing decisions were left to the discretion of the treating cardiology teams. This could have introduced variability in patient management and led to underdiagnosis of acute coronary occlusion although this approach reflects standard clinical practice within the network. We cannot exclude the possibility that a minority of patients with TIMI 2–3 flow had spontaneously reperfused occlusions not classified as ACO. Fourth, only in-hospital mortality was systematically recorded as a clinical endpoint, and other complications were not assessed due to the heterogeneity of final diagnoses, many of which were non-cardiac or non-ischaemic conditions. Additionally, high-sensitivity troponin data were not available for analysis, which precludes assessing the relationship between biomarker status and the decision to perform coronary angiography. Finally, the small sample size of the ACO group (*n* = 28) precluded multivariable analysis to identify independent predictors of acute coronary occlusion. Despite these limitations, the study provides novel insights into cancelled STEMI alerts, underscoring the challenging nature of triage in this complex setting.

## Conclusion

In this prospective study, nearly one-third of STEMI alerts were cancelled following multidisciplinary telematic evaluation, highlighting the increasing role of digital triage tools in optimizing system efficiency. Among cancelled alerts, 4.2% of patients harboured an acute coronary occlusion, a subgroup characterized by a higher burden of cardiovascular risk and frequent presentation with subtle or atypical ECG findings. These results underscore the importance of careful clinical and electrocardiographic assessment when considering cancellation and suggest that further refinement of triage criteria, including risk-based and AI pattern-recognition strategies, may help reduce the risk of missed occlusions while ensuring more efficient use of healthcare resources.

## Data Availability

The data underlying this article cannot be made publicly available due to legal and ethical restrictions related to patient confidentiality. De-identified data may be shared upon reasonable request to the corresponding author and following approval by the relevant institutional ethics committee.
